# Consent Form Reporting on ClinicalTrials.Gov, 2013-2023

**DOI:** 10.1001/jamanetworkopen.2024.18895

**Published:** 2024-06-21

**Authors:** Sydney A. Axson, Reshma Ramachandran, Alexa Lisenby, Nicholas A. Giordano

**Affiliations:** 1 Ross and Carol Nese College of Nursing, Pennsylvania State University, University Park; 2 Rock Ethics Institute, Pennsylvania State University, University Park; 3 Yale School of Medicine, New Haven, Connecticut; 4 Collaboration for Regulatory Rigor, Integrity, and Transparency, Yale School of Medicine, New Haven, Connecticut; 5 Nell Hodgson Woodruff School of Nursing, Emory University, Atlanta, Georgia

## Abstract

This cross-sectional study examines the availability of consent forms for National Institutes of Health–funded trials on ClinicalTrials.gov.

## Introduction

Informed consent documentation is legally, ethically, and scientifically imperative for research and provides prospective trial participants key study information. Historically, consent documents have been difficult to obtain and not consistently publicly available.^1^ As of July 21, 2019, and after a 2-year voluntary period, the revised Common Rule required select federally funded interventional trials to publicly post consent forms no later than 60 days after the last participant visit.^2^ The revision intends to increase research transparency and inform consent development. Although transparency efforts addressing trial registrations and results are well studied, less is known about public availability of consent forms in the context of the revised rule’s recent implementation.^3,4,5,6^ This cross-sectional analysis examined National Institutes of Health (NIH)–funded trial consent form availability on ClinicalTrials.gov.

## Methods

On October 26, 2023, we performed a search for NIH-funded interventional studies with completion dates between August 1, 2013, and August 1, 2023 (ie, a 10-year period of studies with a completion date 60 business days prior), on ClinicalTrials.gov. We exported the resulting 11 202 records. In alignment with the revised rule, only trials with closed recruitment statuses were included (eg, active not recruiting, completed, and terminated). We reviewed records for availability of consent forms and manually extracted the dates forms were posted. Studies were categorized by completion date into 3 periods: prevoluntary, voluntary, and revised rule. The revised rule period refers to when posting for select trials was mandated. The voluntary period refers to the 2-year grace period leading up to the revised rule in which posting of consent was explicitly voluntary and, therefore, distinct from the prevoluntary period. We used a 2-sided *t* test to estimate the difference in the mean annual percentage of studies with consent forms posted before and after the revised rule’s implementation. Statistical analyses were performed using R statistical software version 4.2.3 (R Project for Statistical Computing), and *P* < .05 was considered statistically significant. This study used public, nonidentifiable data that did not constitute human participants research (45 CFR §46.102) and did not require institutional review board review. The study followed STROBE reporting guidelines.

## Results

Of 10 311 trials included in the final sample, 1716 (16.7%) posted consent forms (Table 1). Among studies completed in the prevoluntary period, 105 (2.7%) posted consent forms. The mean percentage of studies posting annually increased from 13.8% during the voluntary period to 30.5% after the revised rule (difference, 16.7 percentage points; 95% CI, 5.6 percentage points to 27.1 percentage points; *P* = .01). Across the 1716 studies with consent forms, the median (IQR) time between study completion and posting was 201.0 (14.0 to 392.3) days, with 617 (36.0%) posting within 60 days (Table 2). The median (IQR) time to posting improved from 1040.0 (378.0 to 2218.0) days in the prevoluntary period to 364.5 (204.0 to 703.5) days during the voluntary period. After the revised rule, median (IQR) time to posting decreased to 103.0 (−14.0 to 346.0) days (Table 2).

**Table 1.** Characteristics of National Institutes of Health–Funded Interventional Studies on ClinicalTrials.Gov, 2013-2023

Characteristic	Studies, No. (%) (N = 10 311)
Consent form posted	1716 (16.7)
Study completion period	
Prevoluntary (before June 28, 2017)	3904 (37.9)
Voluntary period (June 29, 2017, to July 20, 2019)	2052 (19.9)
Revised rule (after July 21, 2019)	4355 (42.2)
Study status	
Active not recruiting	156 (1.5)
Completed	9018 (87.5)
Terminated	1137 (11.0)
Results posted	
No	4974 (48.2)
Yes	5337 (51.8)
Phase	
Early phase 1	263 (4.9)
Phase 1	1355 (25.2)
Phase 1 to phase 2	561 (10.4)
Phase 2	2129 (39.6)
Phase 2 to phase 3	122 (2.3)
Phase 3	590 (11.0)
Phase 4	352 (6.6)
Sample enrollment, median (IQR), No. of participants	60 (25-178)
Intervention type^a^	
Behavioral	3421 (33.2)
Biological	1270 (12.3)
Combination product	23 (0.2)
Device	671 (6.5)
Diagnostic test	59 (0.6)
Dietary supplement	276 (2.7)
Drug	4439 (43.1)
Genetic	66 (0.6)
Other	2883 (28.0)
Procedure	939 (9.1)
Radiation	487 (4.7)
Multiple interventions	3171 (30.8)
Consent forms posted by study completion period	
Prevoluntary (before June 28, 2017) (n = 3904)	105 (2.7)
Voluntary period (June 29, 2017, to July 20, 2019) (n = 2502)	284 (13.8)
Revised rule (after July 21, 2019) (n = 4355)	1327 (30.5)
Consent document length, median (IQR), No. of pages (n = 1716)	10.0 (7.0-15.0)
Consent posted <60 d (n = 1716)	617 (36.0)
Time between study completion and consent upload, median (IQR), d (n = 1716)	201.0 (14.0-392.3)

^a^ Cells do not sum to total because of studies potentially having multiple interventions.

**Table 2.** Consent Form Availability by Regulatory Period

Variable	Total sample	Prevoluntary period	Voluntary period	Revised rule
All studies				
No.	10 311	3904	2052	4355
Studies with a consent form posted, No. (%)	1716 (16.6)	105 (2.7)	284 (13.8)	1327 (30.5)
Studies that posted a consent form within 60 d, No. (%)	617 (6.0)	3 (0.1)	34 (1.4)	580 (13.3)
Studies with consent forms				
No.	1716	105	284	1327
Studies that posted consent form within 60 d, No. (%)	617 (36.0)	3 (2.9)	34 (12.0)	580 (43.7)
Time to post consent forms, median (IQR), d	201.0 (14.0 to 392.3)	1040.0 (378.0 to 2218.0)	364.5 (204.0 to 703.5)	103.0 (−14.0 to 345.0)

## Discussion

In this cross-sectional study examining NIH-funded trials on ClinicalTrials.gov over the course of a decade, consent form availability improved over time, yet time to posting often exceeded 60 days even after the revised rule (Table 2). This may indicate that investigators post consent forms when regulations require results be made available (eg, 1 year after completion). Study limitations include not being able to verify which trials were subject to the revised rule, which studies required participant consent, and whether consent forms were available on other federal websites. However, including only NIH-funded interventional studies mitigates these limitations. Importantly, consent documents narrow trial information down to what is most relevant for prospective participants and is often the only document written for participants. Public availability of consent documents could impact trial recruitment; therefore, making these forms available to prospective participants while trials are still active should be considered.

## Notes

Supplement.
Data Sharing Statement
