# Supplementary material for: Consent Form Reporting on ClinicalTrials.Gov, 2013-2023
Source: JAMA Netw Open. 2024 Jun 21;7(6):e2418895. doi: 10.1001/jamanetworkopen.2024.18895 (PMC11193120; doi:10.1001/jamanetworkopen.2024.18895)
Supplement: Supplement. — Data Sharing Statement [file jamanetwopen-e2418895-s001.pdf]

Data Sharing Statement

Axson. Consent Form Reporting on ClinicalTrials.Gov, 2013-2023. *JAMA Netw Open*.
Published June 21, 2024. doi:10.1001/jamanetworkopen.2024.18895

Data
Data available: Yes
Data types: Data (not involving human participants)
How to access data: Data inquiries should be sent to Dr. Sydney Axson at saaxson@psu.edu
When available: With publication

Supporting Documents
Document types: None

Additional Information
Who can access the data: Approved proposals and with approval of research team
Types of analyses: Specified purposes
Mechanisms of data availability: After approval and with investigator support
